# Automatically Identifying Fusion Events between GLUT4 Storage Vesicles and the Plasma Membrane in TIRF Microscopy Image Sequences

**DOI:** 10.1155/2015/610482

**Published:** 2015-05-19

**Authors:** Jian Wu, Yingke Xu, Zhouyan Feng, Xiaoxiang Zheng

**Affiliations:** ^1^Department of Biomedical Engineering, College of Biomedical Engineering & Instrument Science, Zhejiang University, Hangzhou 310027, China; ^2^Zhejiang Provincial Key Laboratory of Cardio-Cerebral Vascular Detection Technology and Medicinal Effectiveness Appraisal, Hangzhou 310027, China; ^3^Key Laboratory for Biomedical Engineering of Ministry of Education, Hangzhou 310027, China; ^4^Qiushi Academy for Advanced Studies, Zhejiang University, Hangzhou 310027, China

## Abstract

Quantitative analysis of the dynamic behavior about membrane-bound secretory vesicles has proven to be important in biological research. This paper proposes a novel approach to automatically identify the elusive fusion events between VAMP2-pHluorin labeled GLUT4 storage vesicles (GSVs) and the plasma membrane. The differentiation is implemented to detect the initiation of fusion events by modified forward subtraction of consecutive frames in the TIRFM image sequence. Spatially connected pixels in difference images brighter than a specified adaptive threshold are grouped into a distinct fusion spot. The vesicles are located at the intensity-weighted centroid of their fusion spots. To reveal the true *in vivo* nature of a fusion event, 2D Gaussian fitting for the fusion spot is used to derive the intensity-weighted centroid and the spot size during the fusion process. The fusion event and its termination can be determined according to the change of spot size. The method is evaluated on real experiment data with ground truth annotated by expert cell biologists. The evaluation results show that it can achieve relatively high accuracy comparing favorably to the manual analysis, yet at a small fraction of time.

## 1. Introduction

Accurate regulation of insulin is essential for the maintenance of glucose homeostasis in human body. As a member of the protein family of glucose transporters (GLUTs), glucose transporter type 4 (GLUT4) proteins are preliminarily stored within intracellular membrane bound secretory vesicles inside adipose tissues and striated muscle (skeletal and cardiac), also known as GLUT4 storage vesicles (GSVs). Defects in the activity of this protein have been implicated in some forms of insulin resistance and type II diabetes mellitus. When an insulin receptor on cell surface is activated, insulin induces a rapid increase in the uptake of glucose by inducing the translocation of GSVs from intracellular compartments to the plasma membrane. It has long been essential for membrane trafficking to exactly and quantitatively decipher the dynamic behavior of membrane bound secretory vesicles. However, traditional methods from molecular biology and biochemistry are unable to resolve discrete steps of vesicle movement fundamentally [1]. Total Internal Reflection Fluorescence Microscope (TIRFM) can observe layers as thin as 100 nm of a specimen adjacent to the coverslip, making it a widely used tool for observing biological activities near the cell surface, such as endocytosis and exocytosis. Much more quantitative information can be extracted to support biological research through analyzing TIRFM image data. However, it is still a standard practice for most biologists to manually analyze high throughput images generated from* in vivo* observation and visually observe vesicle behaviors. This work is not only time consuming but is also error-prone and nonreproducible, which always induces subjective biases. It is a great need for developing an effective TIRFM image analysis system in biomedical research, which is a novel area in bioimaging, also a subsidiary branch of computing-based image processing [2].

**Figure 1:**
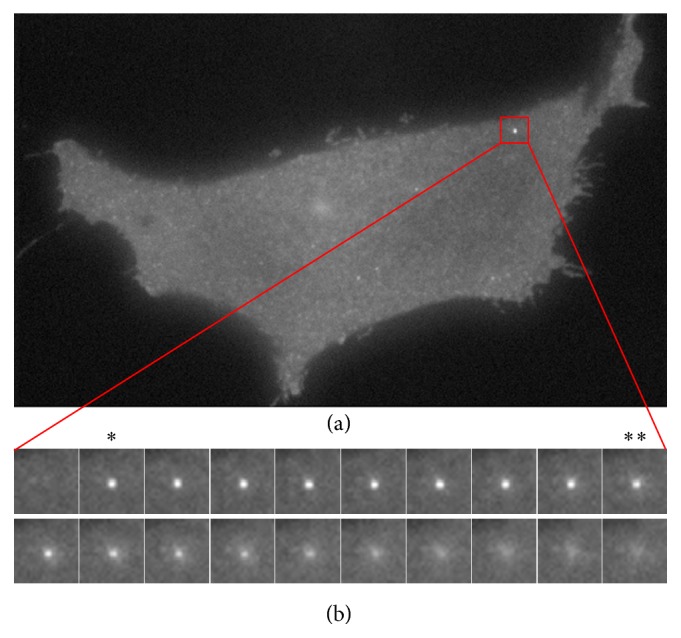
Consecutive time frames from 11 to 30 (b) show that a prominent fusion event corresponds to the patch of interest in a TIRFM image sequence (a). *∗* indicates the fusion pore opening, that is, the initiation of a fusion event. *∗∗* indicates the initiation of a diffusion process. Here, transition time is 1.6 s (8 frames, sampling rate is 5 frames/s).

A fusion event of GSVs comprises final steps of an exocytosis behavior, which includes the processes of fusion pore opening and vesicle diffusion. As the GSVs dock to the plasma membrane, a transient and moderate increase of fluorescence can be observed by TIRFM once the fusion pore of a GSV opens. The vesicles halt and vibrate at the same place for a period (named transition time) and then diffuse away from the fusion site visualized as a fluorescence puff to the cell surface or a small explosion at the cell membrane. GLUT4 is then inserted and becomes the integral membrane (transmembrane) protein. Glucose can be transported into the cell down its concentration gradient in a process called facilitated diffusion. The diffusion process of a fusion vesicle comprises a rapid decrease in fluorescence intensity at the fusion site, a widening of vesicle size and a spreading of signal intensity [3], which is the hallmark for identifying fusion events. A prominent fusion event which comprises fusion pore opening and diffusion process is depicted in Figure 1. While some nonfusion vesicles do not diffuse at the plasma membrane after fusion pore opening, they undock or leave the cell surface and return back into the cell at last.

Little has been done towards the identification of fusion events between GSVs and the cell membrane in TIRFM image sequences. Some of the current existing methods are not fully automated [3–5]. Image processing techniques are usually used to detect the positions of GSVs and sort them out from each frame in an image sequence. Corresponding positions for the same vesicle can be linked to a trajectory of vesicle movement. Before identifying the fusion events, the termination of GSVs trajectories (named death events) should first be located. Subsequently, each single-vesicle trajectory is screened for a possible fusion event primarily based on rules, which derived from quantitative characterization of manually identified fusion process. In Vallotton et al.'s [6], a fully automated system was designed for fusion events detection based on vesicle tracking and rigid template matching. On the basis of Vallotton's study, Mele et al. proposed an improved one, where each fusion candidate was described by a set of novel domain specific descriptors. Similarity scores between genuine fusion events (prototype events, set manually by an expert) and fusion candidates were calculated in the Principle Component Analysis (PCA) eigenspace for fusion identification [2]. The identification of fusion events, which takes the multiple vesicles tracking into account, presents a significant hurdle to surmount. A trajectory may end when a vesicle simply undocks from the plasma membrane, when two vesicles fuse together or when the trajectory is built erroneously, usually resulting in inaccurate location of death events. A strong dependence on the standard fusion template makes finding a fitted correlation kernel for different types of fusion events a nigh impossible task. Based on a usually unsatisfied assumption that a vesicle remains stationary for around *N* frames before it fuses or undocks, a novel approach is proposed in [7]. This method detects fusion and undocking events by first searching for docked vesicles that “appear” to and “disappear” from the field of view, then uses a diffusion model to classify them as either fusion or undocking events.

## 2. Method Outline

In this paper, a fully automated fusion events identification system is proposed, which comprises detecting fusion pore opening and fusion site of fusion vesicles, characterizing the fusion process by Gaussian fitting, and finally identifying the fusion events according to size change of fusion spots. To precisely detect fusion pore opening and fusion site, GSVs are labeled with VAMP2-pHluorin, which is a pH-sensitive reporter. The pH sensitivity of pHluorin has been exploited to visualize the fusion pore opening [1]. When vesicles dock to the cell surface and fusion pore opens, VAMP2-pHluorin is expressed as a transmembrane protein. The sudden rise in fluorescence can be observed due to the different pH value between the inside and outside of the cell. According to this apparent change in fluorescence, we employ the moving average differentiation instead of absolute differentiation between two consecutive frames to identify the initiation of fusion events. To derive the fusion site, an adaptive threshold more commonly known as the Mean Absolute Deviation (MAD) is applied to reduce noise saturated points caused by small variations and other artifacts in difference images. This threshold has proven to be useful when compared to a biologist's visual identification in this experiment. The assumption given in this paper is that no other fusions occur at the same place where a fusion process already exists. Because vesicles do not exhibit much movement upon docking to the cell surface, a square patch of image sequence with each candidate vesicle in its center is cropped for further analysis. Two-dimensional (2D) Gaussian models are used to derive the size of fusion spots during the fusion process. Depending on 2D Gaussian fitting, the total intensity of a fusion spot, intensity at fusion site (intensity weighted centroid of a fusion spot) can also be calculated. The method is evaluated on real data with ground truth annotated by biologists. Evaluation results show that it can achieve relatively high accuracy at a low computation cost.

### 2.1. Detecting the Fusion Candidate Vesicles

In our experiment, due to the benefit of the pH-sensitive reporter (e.g., VAMP2-pHluorin), GSVs cannot be observed until fusion pore opens. The phenomenon that a transient and abrupt fluorescence increases at the cell surface is a strong indicator that vesicles are ready to fuse with the cell membrane. For these apparent changes in intensity, a forward differentiation framework can be used to detect the fusion pore opening. Considering the inherent noise existing in fluorescent imaging, a forward moving average differentiation is used instead of absolute differentiation. In this paper, each difference image Δ*I*_*t*−1_ from *t* = 2 can be achieved by subtracting an iterative background *Acc* from each *I*_*t*_,

(1) Acc=I1for t=2:N  Acc=1−αAcc+αIt  ΔIt−1=It−Accend,

where *N* is the frame number of a TIRFM image sequence; *α* is a user-defined parameter according to the image qualities. In difference images, fusion candidate vesicles correspond to the region where its intensity is higher than local surroundings. To identify these vesicles, an adaptive threshold for difference images called mean absolute deviation (MAD)

(2) $$\begin{array}{c} th=mean\left(abs\left(I_{t}-mean\left(I_{t}\right)\right)\right) \end{array}$$

is used. It has been proven that MAD can extract regions that represent real candidates, as well as eliminate interferences that come from the subtle appearance of fluorescent spots [8]. Sometimes regions corresponding to a same vesicle can be derived many times according to threshold *th* in the difference image sequence. To ensure that there are no other fusion events occurring at the site where a vesicle fusion process already exists, the first difference image of fusion candidate vesicles is considered.

### 2.2. Gaussian Fitting for Fusion Process

After detecting fusion candidate vesicles, further dynamic behaviors can be tracked and deciphered for fusion events identification. According to [2], many cues are used to identify fusion events, such as the coefficient of increased intensity, peak difference, and coefficient of maximum increase. In this paper, the vesicle's total intensity, intensity at weighted centroid, and the fusion size during fusion process are taken into consideration. A fusion event can be described as a transient behavior of intensity and size. During the transition time, there is not much change in the intensity and size of a vesicle. After a short period, the fluorescence of the vesicle rapidly diffuses into the background while the vesicle size increases in a fusion event.

For each of the previously detected fusion candidate vesicles, a patch image sequence centered on a weighted centroid with the extent of (2*w* + 1)×(2*w* + 1) is spatially and temporally cropped from the original TIRFM image sequence. The user defined parameter *w* is an integer larger than a single vesicle's radius and smaller than the nearest distance between vesicles. The extent of an image patch should include the whole vesicle spot and ensure enough space for fluorescence diffusion. Rather than an extensive analysis of the entire image sequence, it can heavily reduce the computation cost.

According to the Point Spread Function (PSF) of microscope systems, vesicles appear as symmetric and round spots in images. The intensity distribution of a vesicle is well approximated by a 2D isotropic Gaussian function using a simplex algorithm with a Least Mean Square Errors (LMSE) estimator [9]. The equation of a 2D Gaussian surface is of the general form

(3) $$\begin{array}{c} G_{w}\left(x,y\right)=I_{c}exp\left[-\frac{{\left(x-x_{c}\right)}^{2}+{\left(y-y_{c}\right)}^{2}}{2{\sigma_{xy}}^{2}}\right], \end{array}$$

where (*x*_*c*_, *y*_*c*_) is the coordinate of a weighted centroid, *I*_*c*_ is the intensity at (*x*_*c*_, *y*_*c*_), and the variance *σ*_*xy*_ is dependent on the actual radius of a vesicle spot, ranging from 0 to *w*. The local background of each frame is subtracted and all pixel intensity values are normalized. The local background for a patch image is approximated using a boxcar average over a square region with the extent of (4*w* + 1)×(4*w* + 1) in the original image sequence:

(4) $$\begin{array}{c} B\left(x,y\right)=\frac{1}{{\left(4w+1\right)}^{2}}\overset{2w}{\underset{i=-2w\;}{\sum }}\overset{2w}{\underset{j=-2w}{\sum }}A\left(x+i,y+j\right). \end{array}$$

The Full Width at Half Maximum (FWHM) is considered as the vesicle radius *r* and divided by *σ*_*xy*_ is approximately the constant 1.1774. The termination of vesicle's movement can be defined when the value of *σ*_*xy*_ multiplied by 1.1774 is bigger than parameter *w*, using the result of directly fitting the 2D Gaussian function to each patch image without local background subtraction.

To eliminate the abrupt interference due to fluorescence turbulence, the integrated average of vesicle radii at each time step is used to quantify the size of the fusion spots. All fluorescence is assumed to be membrane-embedded in either vesicles, internal membrane structures, or the plasma membrane [2]. A vesicle can also be assumed as an isotropic ball with radius *r* when docking to the cell membrane before diffusion. The surface area of a sphere with radius *r* is determined using the following formula area = 4*πr* ^2^. When a vesicle fully fuses with the plasma membrane, the membrane-embedded fluorescence is released to the cell membrane. The radius of the final diffusion spot is at least two times bigger than spot radius before diffusion. According to these, during diffusion process a fusion event can be exactly deciphered if vesicle spot radius is two times bigger than its integrated average at diffusion initiation.

## 3. Experiment and Evaluation Results

In this experiment, VAMP2-pHluorin positive 3T3-L1 adipocytes are imaged using an IX-70 inverted TIRFM microscope (Olympus), which is equipped with both argon (488 nm) and argon/krypton (568 nm) laser lines (Melles Griot), a 60 × 1.45 N.A. oil immersion objective lens (Plan-ApoN; Olympus), and a TIRFM condenser. The TIRFM images were detected with a sampling frequency of 5 Hz with a back-illuminated Andor iXon887 EMCCD camera (1024 × 1024, pixel spacing 0.18 *μ*m, 16 bits; Andor Technologies).

**Figure 2:**
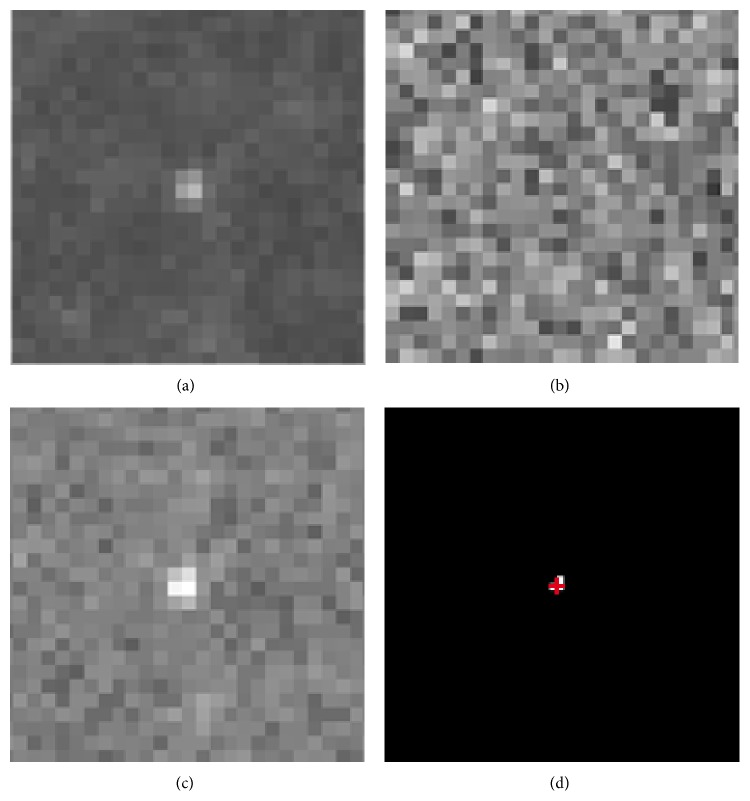
The process of detecting a fusion candidate vesicle. (a) A patch of image with a fusion candidate vesicle in. (b) The absolute differentiation of consecutive images results in a noisy difference image, from which the vesicle is hard to detect. (c) The forward moving average differentiation can achieve a high SNR difference image. (d) A vesicle spot mask derived from (c) with the MAD threshold, which can be used to calculate the intensity-weighted centroid (a red cross).

In this paper, three real TIRFM image sequences, each has 400 consecutive frames, are chosen to evaluate the performance of the proposed method. All image sequences are well annotated by expert cell biologists. During the detection of fusion candidate vesicles, we choose the user-defined parameter *α* = 0.2 for the purpose of removing irrelevant background noise and keeping the vesicle objects. As shown in Figure 2, the forward moving average differentiation can achieve a high signal-to-noise ratio (SNR) difference image comparing to the absolute differentiation method. Without initiation, the adaptive threshold MAD can heavily suppress uneven local background and extract vesicle spot masks from difference images. The intensity-weighted centroid of a fusion vesicle can be calculated using the mask imposed on the original image. The evaluation results of detecting fusion candidate vesicles in three image data sets are shown in Table 1. The detection method combined with the forward moving average differentiation and an adaptive threshold MAD can achieve a relatively high accuracy with both recall and precision up to 90%. Due to the strong noisy image background, some subtle vesicles are missed. While some artifacts suddenly appeared in cell images are mistakenly detected as vesicles through image differentiation step.

**Figure 3:**
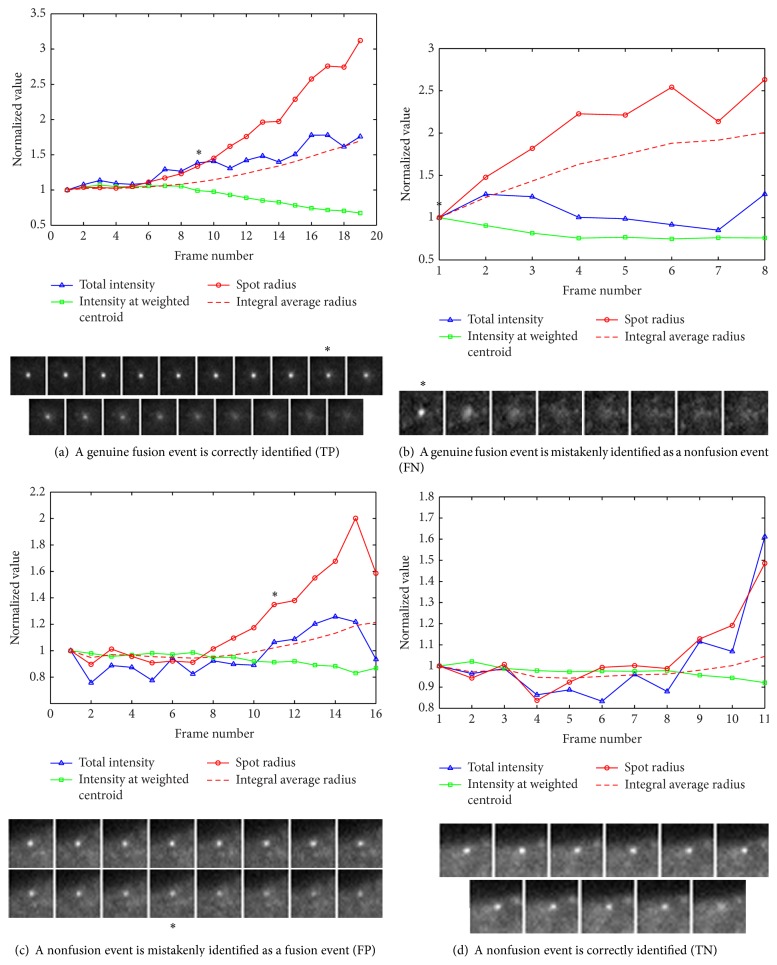
Four typical identification results compare to ground truth. Relationship between the variety of total instensity, intnesity at weighted centroid, and spot radius during vesicle movement. The integral average radius is also calculated (red dashed line) as a threshold for identifing diffusion initiation (>1.2 times, black asterisk) and fusion event (>2 times). (c) A nonfusion at the edge of cell is mistakenly identified as a fusion event, mainly due to unsymmetrical background. All values are normalized to the initiation of fusion pore opening.

According to the image data, the parameter *w* = 10 is taken, because the largest radius of fusion spot is not bigger than 10 pixels. A sequence of patch images with extent of 21 × 21 for each fusion candidate vesicles is cropped. After Gaussian fitting for each frame, the vesicle radius (FWHM of a Gaussian function), intensity at weighted centroid (peak intensity), and total intensity can be derived. The relationship between them during vesicle movement is shown in Figure 3. The integral average radius is also calculated to detect the initiation of the diffusion process, marked with black asterisk. In this paper, the initiation of the diffusion process can be defined at the time when vesicle size is 1.2 times bigger than the integral average size. It means that the size change of a vesicle is not more than 20% within fusion pore duration. The candidate vesicle can be defined as a fusion one if the largest radius of vesicle spot during the diffusion process is 2 times bigger than the integral average size of the vesicle at the initiation of the diffusion. As shown in Figure 3, the assumption in [2, 6] that the integrated intensity over the entire surface is constant is not satisfied due to the consequence of the cells releasing fluorescent materials to the cell surface. The evaluation results for automatically identifying fusion events in three image data sets are shown in Table 2. Four typical identification results comparing to ground truth are shown in Figure 3. Many fusion events mistakenly identified (FP) by this method are at the edge of cell, shown in Figure 3(c). Although local background is reduced, 2D Gaussian fitting cannot output satisfied results due to uneven image background. While the missed one (FN) is due to the rigorous condition, the largest radius of fusion spot during the fusion event is 2 times bigger than the integral average size of the vesicle at the initiation of the diffusion event, shown in Figure 3(b). This method is quite dependent on precise 2D Gaussian fitting. For total image date sets, the evaluation results in Table 2 show that it can achieve relatively high accuracy compared favorably to the manual analysis, yet at a small fraction of time.

## 4. Conclusion

In this paper, we have proposed a fully automated and easily applied method for identifying fusion events. We have evaluated the method using real image data annotated by biologists. Evaluation results show that the forward moving average differentiation combined with an adaptive threshold MAD is more useful for detecting fusion candidate vesicles combined with a pH-sensitive reporter, while 2D Gaussian fitting of fusion process helps to annotate the fusion event in TIRFM image sequences.

## Figures and Tables

**Table 1:** Recall and precision results of detecting fusion candidate vesicles.

	Fusion candidate vesicles ground truth (TP + FN)	Fusion candidate vesicles detected (TP + FP)	Mistakenly detected (FP)	Missed (FN)	Recall	Precision
Sequence 1	131	125	2	8	93.9%	98.4%
Sequence 2	144	140	1	5	96.5%	99.3%
Sequence 3	179	173	5	11	93.9%	97.1%

Average					94.8%	98.3%

**Table 2:** Results of automatically identifying fusion events.

	Fusion events ground truth (TP + FN)	Fusion events detected (TP + FP)	Mistakenly identified (FP)	Missed (FN)	Recall	Precision	False +ve
Sequence 1	47	42	6	11	76.6%	85.7%	7.1%
Sequence 2	62	59	7	10	83.9%	88.1%	8.5%
Sequence 3	89	83	9	15	83.1%	89.2%	10.0%

Average					81.2%	87.7%	8.5%
